# Bibliometric analysis of real-time PCR-based pathogen detection in plant protection research: a comprehensive study

**DOI:** 10.3389/fpls.2023.1129714

**Published:** 2023-06-06

**Authors:** Priyanka Lal, Rahul Kumar Tiwari, Awadhesh Kumar, Muhammad Ahsan Altaf, Abdulaziz Abdullah Alsahli, Milan Kumar Lal, Ravinder Kumar

**Affiliations:** ^1^ Department of Agricultural Economics and Extension, School of Agriculture, Lovely Professional University, Phagwara, India; ^2^ ICAR-Central Potato Research Institute, Shimla, Himachal Pradesh, India; ^3^ ICAR-National Rice Research Institute, Cuttack, Odisha, India; ^4^ School of Horticulture, Hainan University, Haikou, China; ^5^ Botany and Microbiology Department, Faculty of Science, King Saud University, Riyadh, Saudi Arabia

**Keywords:** diagnostic, fungi, bibliometric, on-site detection, infection, PCR

## Abstract

**Introduction:**

The discovery of RT-PCR-based pathogen detection and gene expression analysis has had a transformative impact on the field of plant protection. This study aims to analyze the global research conducted between 2001 and 2021, focusing on the utilization of RT-PCR techniques for diagnostic assays and gene expression level studies. By retrieving data from the 'Dimensions' database and employing bibliometric visualization software, this analysis provides insights into the major publishing journals, institutions involved, leading journals, influential authors, most cited articles, and common keywords.

**Methods:**

The 'Dimensions' database was utilized to retrieve relevant literature on RT-PCR-based pathogen detection. Fourteen distinct search queries were employed, and the resulting dataset was analyzed for trends in scholarly publications over time. The bibliometric visualization software facilitated the identification of major publishing journals, institutions, leading journals, influential authors, most cited articles, and common keywords. The study's search query was based on the conjunction 'AND', ensuring a comprehensive analysis of the literature.

**Results:**

The analysis revealed a significant increase in the number of scholarly publications on RT-PCR-based pathogen detection over the years, indicating a growing interest and investment in research within the field. This finding emphasizes the importance of ongoing investigation and development, highlighting the potential for further advancements in knowledge and understanding. In terms of publishing journals, Plos One emerged as the leading journal, closely followed by BMC Genomics and Phytopathology. Among the highly cited journals were the European Journal of Plant Pathology, BMC Genomics, and Fungal Genetics and Biology. The publications with the highest number of citations and publications were associated with the United Nations and China. Furthermore, a network visualization map of co-authorship analysis provided intriguing insights into the collaborative nature of the research. Out of 2,636 authors analyzed, 50 surpassed the level threshold, suggesting active collaboration among researchers in the field.

**Discussion:**

Overall, this bibliometric analysis demonstrates that the research on RT-PCR-based pathogen detection is thriving. However, there is a need for further strengthening using modern diagnostic tools and promoting collaboration among well-equipped laboratories. The findings underscore the significance of RT-PCR-based pathogen detection in plant protection and highlight the potential for continued advancements in this field. Continued research and collaboration are vital for enhancing knowledge, developing innovative diagnostic tools, and effectively protecting plants from pathogens.

## 1 Introduction

The technology to visualize polymerase-chain-reaction (PCR) products in real time in an ongoing reaction has revolutionized the field of plant pathogen diagnostics (Donoso and Valenzuela, 2018). During the last two decades, the utilization of real-time PCR techniques has dramatically increased and has been extensively popularized among various stakeholders in plant and animal sciences (Buja et al., 2021). Real-time PCR differs from conventional PCR in that the amplified PCR product is measured at every cycle of the PCR process. In actuality, a fluorochrome included within the freshly generated PCR result emits light that is captured by a video camera (Kralik and Ricchi, 2017). Real-time PCR thus enables the amplification to be followed in real time during the exponential phase of the run, enabling precise estimation of the starting material amount. In contrast to end-point PCR methods, the result is not dependent on the reaction’s saturation plateau, which results in erroneous quantification (Kainz, 2000). Real-time PCR has a variety of advantages over other well-known laboratory procedures, making it the method of choice for several sorts of research. It enables the quick, accurate, and extremely sensitive detection of a specific nucleic acid target as compared with other currently available approaches (Kumar et al., 2017; Kumar et al., 2021). Additionally, it allows for the initial target’s absolute quantification. Real-time PCR’s dependability has never been questioned to this point. Additionally, real-time amplification recording saves time and effort by avoiding the need to collect samples at various stages of the PCR experiment. Additionally, some machines can handle queuing plates for up to 24 h straight while processing 384-well plates (Gachon et al., 2004), which may be an advantage for high-throughput research or if quick sample processing is needed (Kumar Tiwari et al., 2019; Kumar et al., 2020; Kumar et al., 2022).

When just a tiny percentage of the sample contains the mutation, real-time PCR provides for quantitative genotyping, detection of single-nucleotide polymorphisms, allelic discrimination, and genetic variations (Zhou et al., 2001; Morlan et al., 2009). The limitations of traditional PCR methods for live systems have become apparent, and there is an increasing need for more advanced techniques. As a result, the use of multiplex PCR methods has become increasingly important in recent years. These techniques involve the use of coupled probes and primers, which are targeted to specific sequences that are relevant to plant/microbe relationships. This approach enables the simultaneous amplification of multiple target sequences in a single reaction, allowing for the efficient and accurate detection of multiple organisms or gene variants. Overall, the use of multiplex PCR methods represents an important advance in the study of live systems and has the potential to provide valuable insights into the dynamics of plant/microbe interactions (Elnifro et al., 2000; Palka-Santini et al., 2009). The development of bioscience over the past century has aided in a thorough understanding of information pertaining to the network of diverse gene modules that interact and carry out integrated cellular function in a coordinated but somewhat isolated manner, or the molecular mechanism of phenotypic expression of genotype (Costanzo et al., 2019). Our understanding of the intricate connections between enzymes, signaling molecules, and numerous small molecules remains incomplete, and a considerable portion of the genome’s functionality remains a mystery. To comprehensively understand how metabolism is regulated, we need to gain more insight into gene expression, DNA recognition by proteins, transcription factors, and the mechanisms of action of various drugs and small molecules. Such knowledge is crucial for developing effective strategies to manipulate metabolic processes to improve human health and combat diseases. As research continues in these areas, we can expect to uncover new insights into the complex networks governing cellular metabolism (Bai et al., 2013). The link between ecologically influenced or disease-related phenotypes and cellular expression patterns has been extensively studied using gene expression profiles. In-depth knowledge of the biology of plant/microbe interactions, particularly concerning the ecology, etiology, and epidemiology of plant pathogenic microorganisms, is being provided by PCR-based detection technologies using species-specific primers (Hariharan and Prasannath, 2021).

Since the earliest study by Böhm et al. (1999), the number of real-time PCR tests designed to measure the extent of plant infection by a pathogen has increased (Böhm et al., 1999). The majority of them rely on two distinct plant and pathogen DNA sequences being relatively quantified. Compared with conventional protocols based on symptom recording or conidiophore or colony counts, they are quicker, more precise, and more sensitive. Most importantly, they can be applied to almost all pathosystems. Due to these factors, they are frequently utilized for both practical and field-based disease diagnosis (Liu et al., 2019). These assays have been extensively used in the field of plant pathology due to their high sensitivity, specificity, and rapid detection capabilities. They are particularly useful for detecting low-level infections and for identifying new or emerging pathogens that can cause devastating crop losses. The application of real-time PCR-based assays has revolutionized the diagnosis and management of plant diseases, enabling growers to respond to outbreaks and implement effective control measures quickly (Mirmajlessi et al., 2015; Londoño et al., 2016; Tiwari et al., 2020a; Tiwari et al., 2020b). Furthermore, real-time quantitative PCR has emerged as the preferred technology for determining food adulteration or contamination. Compared with ELISA, PCR tests are simpler to create because they do not need to be developed with particular antibodies. Because DNA is more thermo-stable than proteins, PCR tests offer greater sensitivity and are more suited for the detection of undesirable dietary constituents in highly processed foods. For instance, a real-time PCR test of cereal genes can be used to control the absence of gluten in infant food (Sandberg et al., 2003). Real-time quantitative PCR has also been demonstrated to be an excellent method for detecting adulteration of durum wheat pasta with common wheat (*Triticum aestivum*) (Terzi et al., 2003).

The validation of data produced from microarray investigations is one of the fastest-growing uses of real-time PCR (Deepak et al., 2007). The validity of microarray experiments can occasionally be put into question. Cross-hybridization between cDNA representatives of gene family members on cDNA-based chips may produce inaccurate results since plants exhibit a large number of multigene families (Palka-Santini et al., 2009). However, compared with real-time PCR, which is frequently restricted to fewer genes, microarray assays can analyze thousands of genes in a single step. Because real-time PCR devices can detect only a finite number of fluorophores and light spectra, only a few genes can be detected in a single multiplex PCR run. Real-time PCR necessitates the construction of individual oligonucleotides for each gene to be analyzed. Consequently, a common approach is to identify a small number of potentially relevant genes using microarray tests and then to confirm those candidates using real-time RT-PCR analysis (Kumar et al., 2020).

Through a quantitative examination of patterns in the body of scientific literature, bibliometrics enables the identification of new trends and knowledge structures in the research subject (Fan et al., 2020; Lin et al., 2020; Akintunde et al., 2021; Dmytriw et al., 2021). Based on published studies on statistical data on plant pathogen detection, one may comprehend the general global picture, including the number of such studies, the level of research capability in various nations, the major research institutions, the top journals publishing such studies, and other factors (Tang et al., 2022). To develop a comprehensive scientific research strategy, it is crucial to understand the current state of research, key areas of focus, and significant gaps in knowledge. In this context, a bibliometric analysis can provide an accurate, validated, and systematic overview of developments in plant-based pathogen detection technologies. This analysis can help identify the most recent detection techniques and their efficacy and pinpoint research boundaries, topic trends, and innovative collaborations between scientists at various universities. By conducting this analysis, we can gain insights into the current state of research in this area, identify knowledge gaps, and develop strategies to address them. Overall, bibliometric analysis is a powerful tool for understanding the research landscape, which is essential for developing a focused and effective scientific research strategy.

## 2 Material and methods

**Figure 1:**
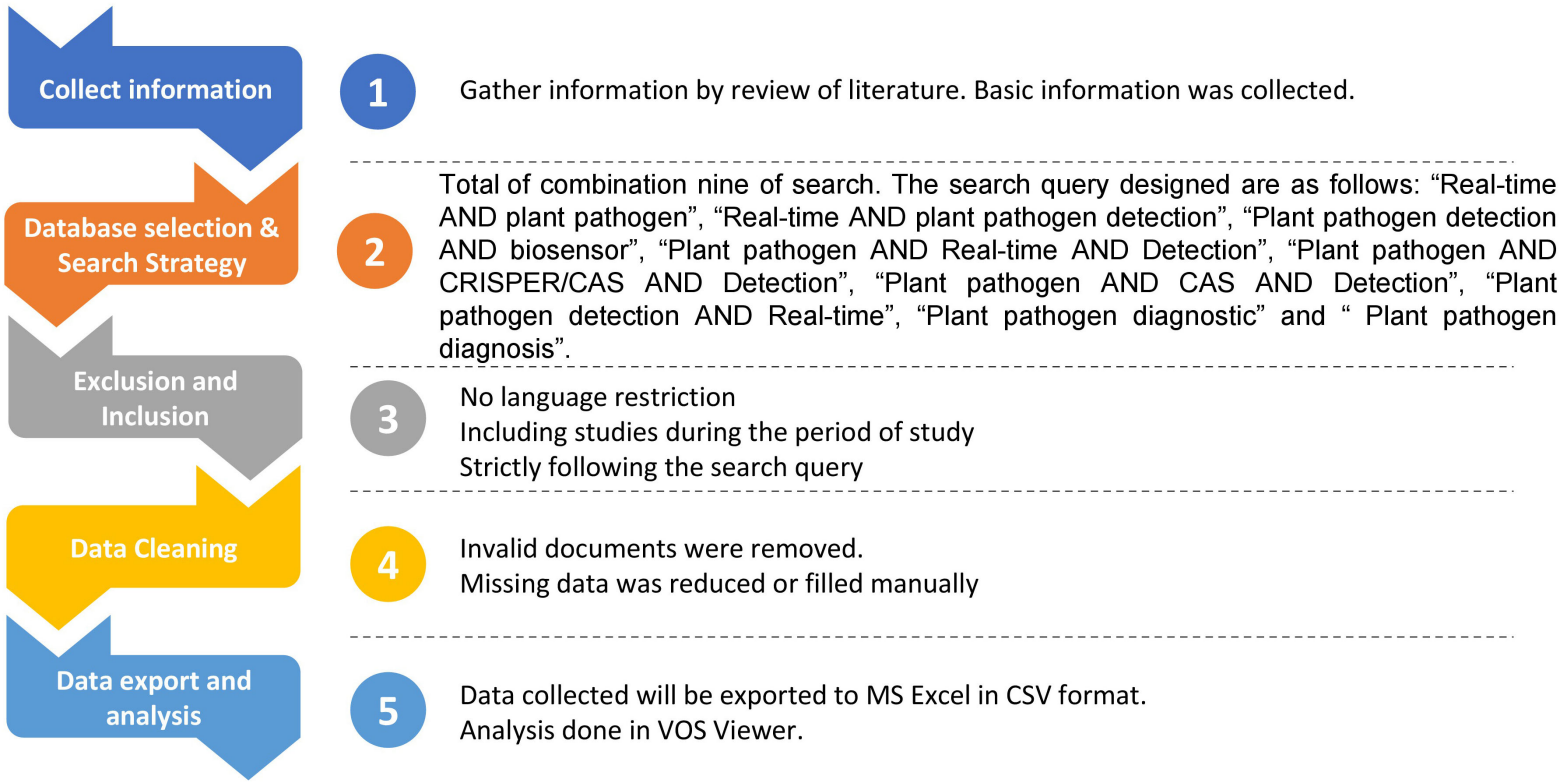
Flow chart of bibliometric analysis.

We acquired pertinent “Dimensions” data for the study using several search engines. Dimensions offer a diverse database of research outcomes. The platform enables stakeholders to get data and construct concepts simply. Researchers are now using bibliometric methods to track and analyze the impact of the pandemic on various scientific disciplines and to identify emerging research areas related to COVID-19. This approach has helped to inform public health policies and guide research priorities in the fight against the pandemic. Overall, the COVID-19 crisis has highlighted the importance of real-time bibliometric studies in providing timely and relevant insights into the rapidly evolving research landscape (Hook et al., 2021). Dimensions enables scholars to search full-text data for various years and scholarly works like pre-prints, articles, chapters, conferences, monographs, and edited books. This expanded data collection for examination opens up new research opportunities. Machine learning techniques have become increasingly popular in constructing links between items in large datasets, enabling researchers to identify patterns and relationships that would be difficult to detect manually. Per-object categorizations and person and institution disambiguation provide further context, improving the accuracy and reliability of results. These tools allow researchers to extract valuable insights from complex datasets, revealing hidden connections and trends that may have been overlooked in traditional analysis methods, ultimately enabling more informed decisions based on data-driven insights. The detailed flow chart of the steps followed in the study is depicted in **Figure 1**.

### 2.1 Database selection and search query

In this investigation, the Dimensions database provides details on the authors’ names, title of the article, author’s affiliation, etc., making it appropriate for bibliometric research. The data extracted possess numerous analytical functions, including citation and subject analysis. There were no restrictions on language, and the data extracted were in English as the search query required an English title and abstract, allowing researchers to authenticate the substance of non-English documents by title/abstract content. A correct search query is an essential component of a bibliometric study in order to obtain high-quality and dependable findings. To form the query, the initial idea was formed after reading scientific papers and identifying research gaps. The study’s search query was based on the conjunction “AND.” A total of 14 combinations of search queries were used in order to arrive at the final data. The search queries designed are as follows: “Real-time AND plant pathogen,” “Real-time PCR and plant pathogen detection,” “Cycle threshold AND plant pathogen,” “RT-RPA and plant pathogen,” “Lateral Flow devices AND plant pathogen,” “LAMP-based detection,” “Real-time AND plant pathogen detection,” “Plant pathogen detection AND biosensor,” “Plant pathogen AND Real-time AND Detection,” “Plant pathogen AND CRISPER/CAS AND Detection,” “Plant pathogen AND CAS AND Detection,” “Plant pathogen detection AND Real-time,” “Plant pathogen diagnostic,” and “Plant pathogen diagnosis.” Later the data derived were merged.

### 2.2 Document exclusion and inclusion

The data were extracted strictly according to the search query because any departure could result in erroneous results. The Dimensions database provides articles in several categories such as articles, books, notes, and pre-prints. We included only research articles and left out the rest of the categories. The study period was limited to the last 20 years (2001–2022). The data were collected in a single day to eliminate ambiguity in the results caused by the daily addition of documents. Because the search query was in English, all of the documents were in the English literature.

### 2.3 Data extraction and analysis

The data were obtained in the form of an MS Excel file in CSV format for further research. The Excel software was used for tabular analysis, and the VOSviewer software was used for map visualization (van Eck and Waltman, 2011). The citation value can be utilized for analysis in order to assess the scientific contribution and influence of publications. The “connection strength” metric derived from visualization maps was used to assess international research collaboration among participating countries. The strength of any two countries’ connections is a measure of the degree of their scientific collaboration. The strength of the link varies according to the thickness of the connecting wires between countries. The more the research collaborations, the greater the importance of link strength, or the thickness of the connecting wire.

## 3 Results and discussion

The data were analyzed, and the following estimates were made. Various forms of analysis were performed, including citation analysis and authorship analysis. **Table 1** shows the results of a citation analysis performed to understand the influence of publication sources better. *Plos One* published the most papers and received the most citations during the research period, followed by *BMC Genomics* and *Phytopathology*. However, a similar pattern was not observed when calculating citations per paper. The *European Journal of Plant Pathology* received the most CPP citations, followed by *BMC Genomics* and *Fungal Genetics and Biology*.

**Table 1:** Top 20 sources where the documents were published during the study period.

Source	Documents	Citations	Citations per paper
*Plos One*	29	1,188	40.97
*BMC Genomics*	15	707	47.13
*Phytopathology*	7	692	98.86
*European Journal of Plant Pathology*	15	466	31.07
*Plant Disease*	24	425	17.71
*Molecular Plant-Microbe Interactions*	10	422	42.20
*Applied and Environmental Microbiology*	9	382	42.44
*Canadian Journal of Plant Pathology*	8	337	42.13
*Journal of Microbiological Methods*	10	329	32.90
*Planta*	11	328	29.82
*Molecular Plant Pathology*	8	305	38.13
*Fungal Genetics and Biology*	6	265	44.17
*Frontiers in Plant Science*	19	234	12.32
*Plant Physiology and Biochemistry*	5	201	40.20
*Frontiers in Microbiology*	8	199	24.88
*BMC Plant Biology*	8	195	24.38
*Plant Pathology*	5	170	34.00
*Fungal Biology*	6	160	26.67

Following data collection, it was analyzed in the VOSviewer, leading us to a list of journals that produced most of the articles throughout the study period. The VOSviewer analysis yielded a total of 28 top journals that met the limitation of a minimum of five documents. **Figure 1** depicts a visual representation of the citation analysis.

**Figure 2:**
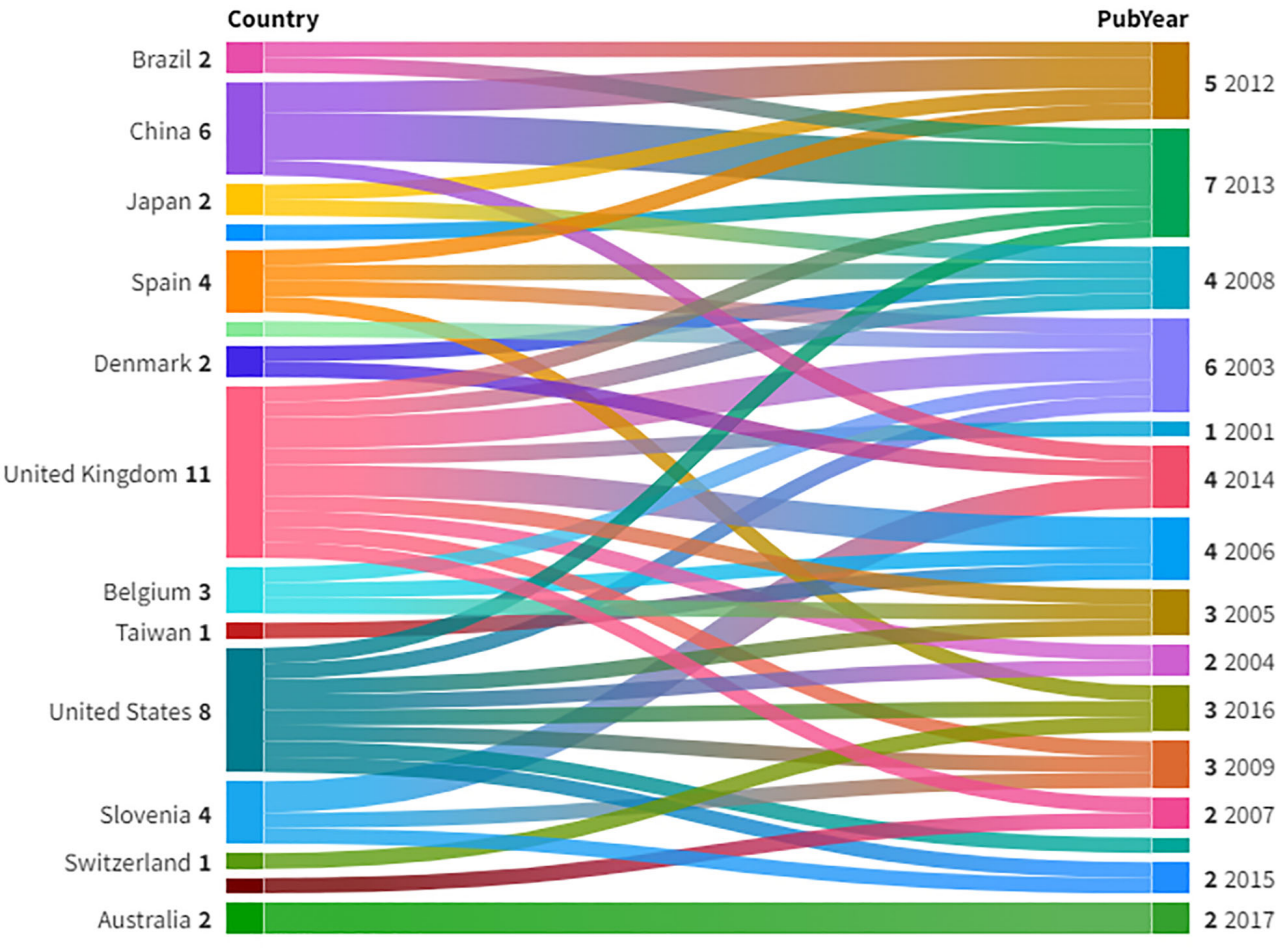
Network map for citation sources.

The analysis found that the articles published during the study period on the real-time detection of pathogens in plants were published in journals like *Plos One*, *Plant Disease*, and *Frontiers in Plant Science* with 29, 24, and 19 documents, respectively. In addition, our selection contained a wide variety of journals. The larger the bubble, the higher the impact of that particular source. It can be seen from **Figure 2** that there is a strong connection between the *Plos One* and *Plant Disease* journals. This depicts the publishing behavior of the researchers on the study theme.

### 3.1 Intellectual structure

**Figure 3:**
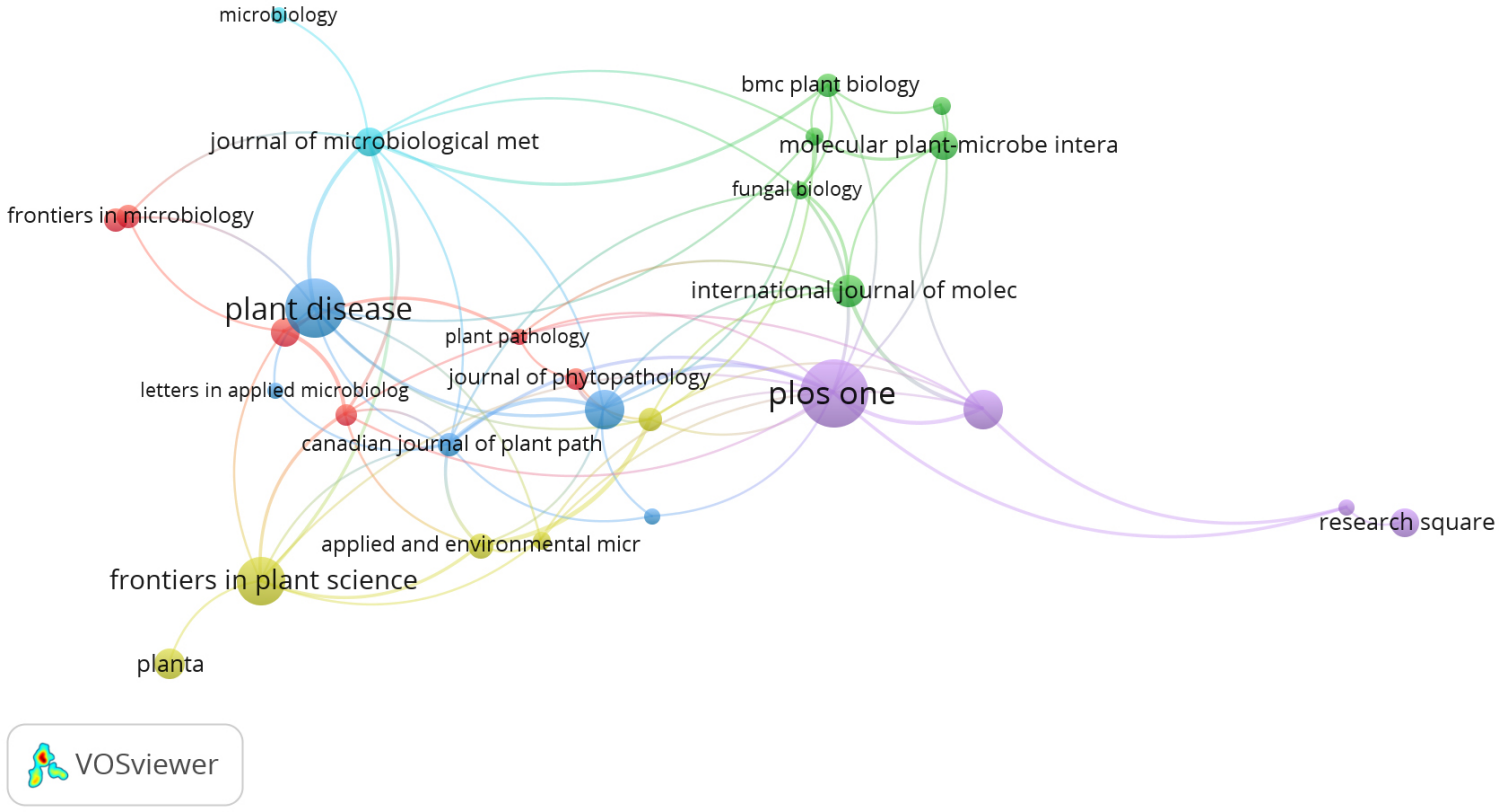
Sankey Graph between countries and publication year. Source: VOSviewer.

The intellectual structure is typically based on the interest of the researcher in a particular topic (Marsilio et al., 2011). In the case of pathogen detection, either the countries with the same pathogen attack collaborate or the countries with possible invasion of foreign virus do. In this paper, we tried to map the intellectual structure of scholars and the concerned countries. **Figure 3** shows a Sankey diagram for the most productive countries and the publication year of the same. The diagram is the representation of how the items are distributed, and the thickness of the link shows the volume of the flow. **Figure 3** shows the top countries with the highest citations and the year in which they published the articles.

### 3.2 Analysis of co-authorship among countries

The normalized citation value of each country’s published documents can be used to compare the scientific impact of publications from different countries (Bornmann and Wohlrabe, 2019). The concept of normalized citation value has been derived from the network visualization maps generated by VOSviewer. In these maps, the size of each node representing a particular country corresponds to its normalized citation value, which is essentially a measure of the impact and quality of its research output. The larger the size of a country’s node in the normalized citation visualization map, the higher its research output’s scientific significance. Therefore, this metric provides a valuable means of assessing the relative performance of countries in terms of research impact and productivity based on their normalized citation values.

**Figure 4:**
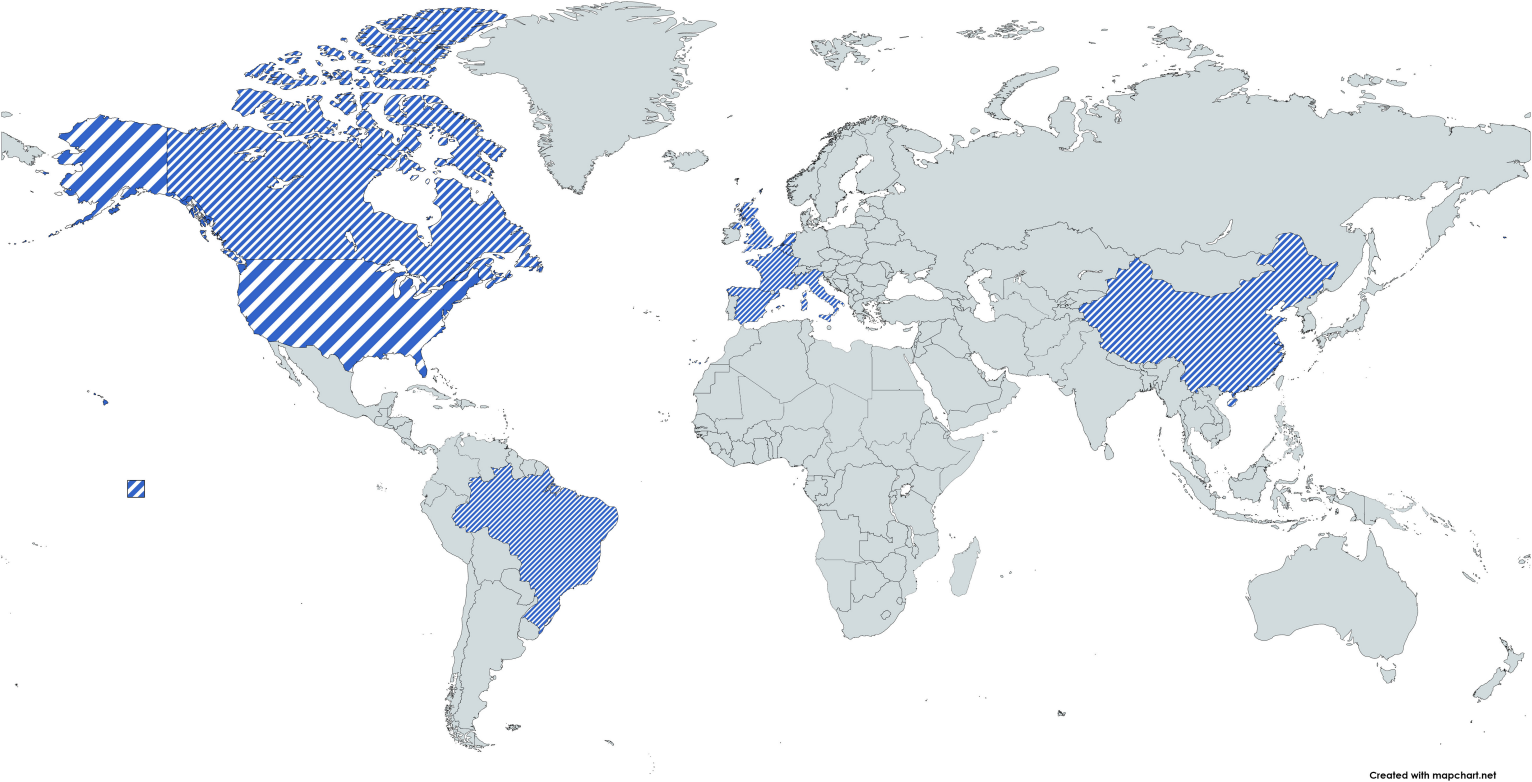
World map of top 10 countries with highest citations.

**Figure 5:**
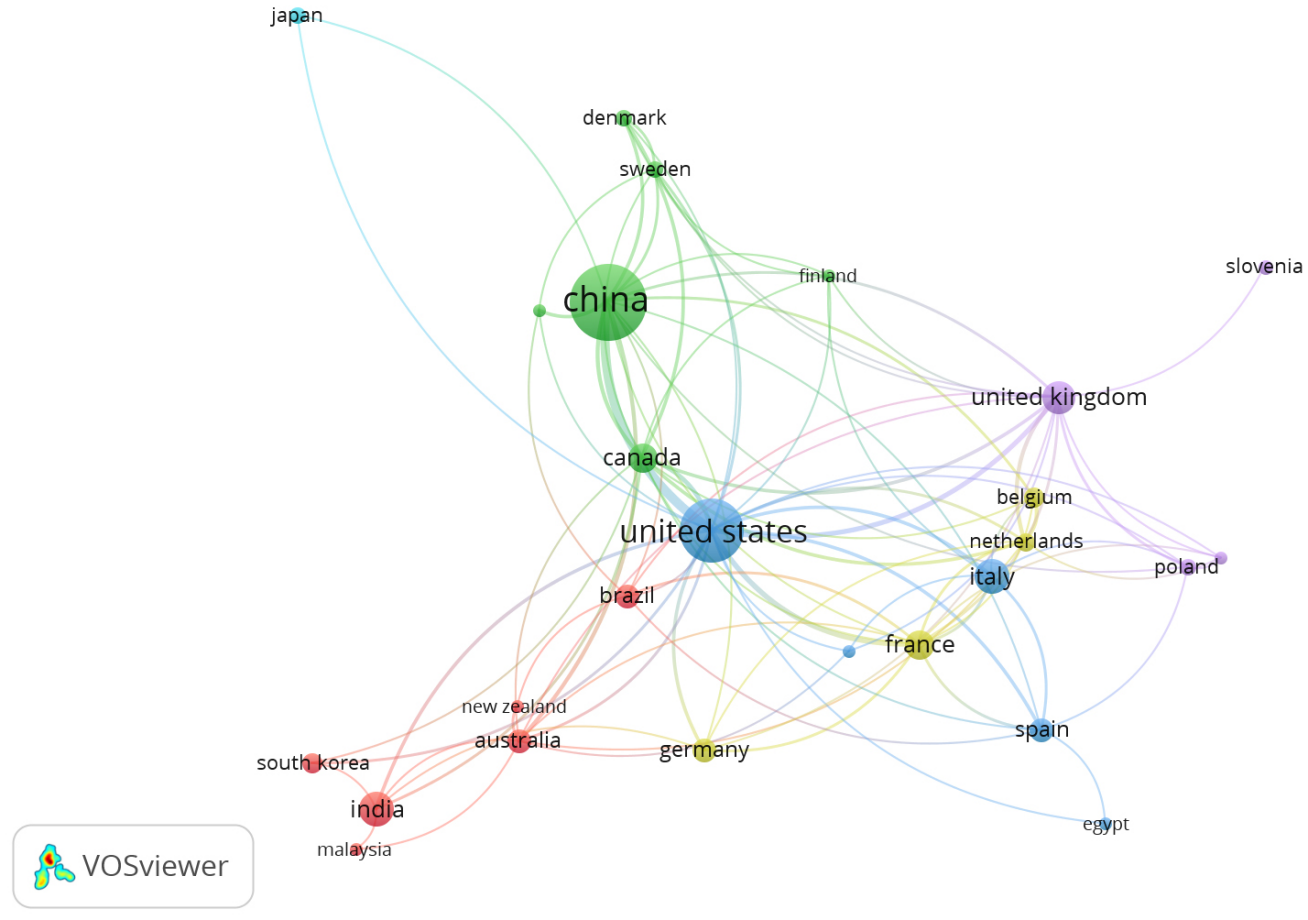
Network Visualization map for Co-authorship countries. Source: VOSviewer.

The data were analyzed in VOSviewer by keeping some constraints such as a minimum number of documents of 5. There was no minimum number of citations kept in this analysis. It was found that out of 56 countries, only 26 meet the threshold. There were six clusters found in the analysis and each cluster had a dominating country in terms of citations. The United Nations and China had publications that had the highest number of citations and publications. The clusters were a group of countries that collaborated with each other. The top 10 countries with the highest number of citations were visualized using a world map, as shown in **Figure 4**. It clearly shows that the countries of interest were among the top 10 cited countries. In **Figure 5**, the line connecting two independent bubbles indicates a relationship in each source of highly referenced documents (van Eck and Waltman, 2011). The complexity represents the significant interconnectedness of the countries, and the differences in the color of the bubbles represent differences in temporal pattern (Zhu et al., 2009).

### 3.4 Co-authorship authors

**Figure 6:**
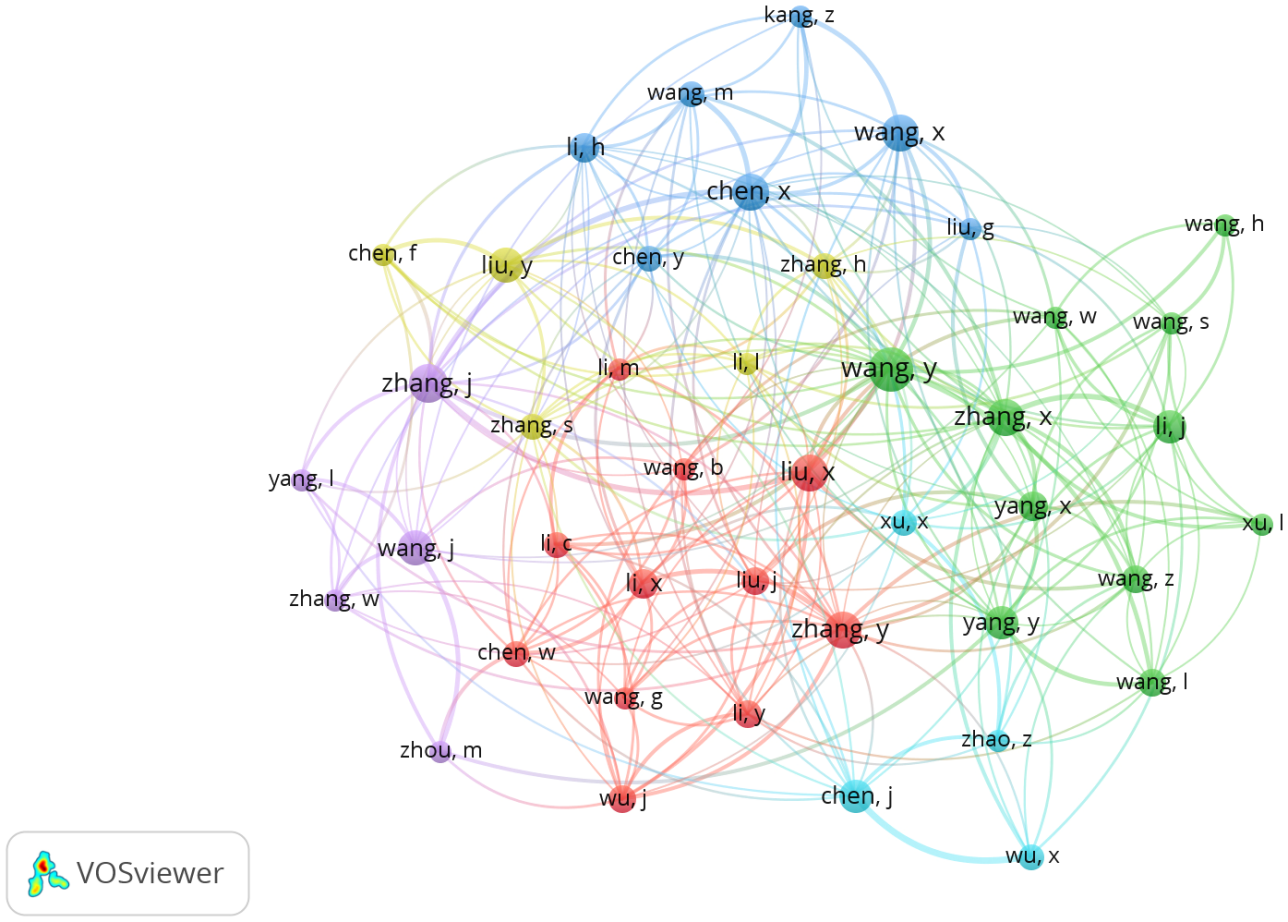
Network visualization map of Co-authorship authors. Source: VOSviewer.

Collaboration has been found to have a generally beneficial effect on citation impact in almost all subject domains and at all levels of aggregation (Moed et al., 1991; Narin et al., 1991). Bibliometrics shows the authors that collaborate with each other as it shows the scientific engagement and relationship between teams and organizations. Such analysis of co-authorship among authors was done using the software and is presented in **Figure 6**. Co-authorship is a valuable tool that enables us to identify, measure, and illustrate the links between individual contributors to a particular piece of research. Through co-authorship, we can gain insight into the extent of collaboration between researchers and track the development of their collaborative relationships over time. Co-authorship can also measure the quality and impact of scientific output, as it indicates the degree to which research is being conducted in partnership with other experts in the field. Overall, co-authorship is a vital tool that helps to foster collaboration and teamwork and is an important element in the production of high-quality scientific output.

In the analysis, we kept the threshold as 100 citations and five documents per author. It was found that out of 2,636 authors, only 50 meet the threshold. There was total of six clusters found in the analysis. Co-authorship occurs when two authors collaborate on a study. It is one of the most prominent and well-documented types of scientific co-operation. Almost every feature of scientific collaboration networks can be accurately tracked by examining co-authorship networks using bibliometric methodologies (Glänzel and Schubert, 2006). These co-operation networks (co-authorship) indicate research teams as well as characteristics that influence collaboration impact or production. The limitation here emphasizes that older papers have more citations than younger documents, which reduces the likelihood of fresh publications being examined and affects the order of the top writers in the list (Luther and Tiberius, 2020; Wu et al., 2021).

We discovered the co-authorship network approach to be the best and most dependable method to adopt in our research methodology based on our research requirements. Interdisciplinary collaboration, on the other hand, has been found to improve research outputs. According to the study, co-authors are two authors who collaborate on research and have the most concrete and well-documented kind of research interaction (Glänzel and Schubert, 2006). Almost any component of scientific collaboration networks can be reliably studied by examining co-”author” networks using bibliometric techniques. These co-authorship co-operation networks demonstrate the impact of co-authorship, research teams, and collaborative output.

### 3.5 Co-citation source analysis

According to Rousseau (1994), the three major uses of co-citation are, namely, qualitative and quantitative appraisal of scientists, publications, and scientific institutions; modeling of the historical development of science and technology; and information search and retrieval. In other words, using journal relationships, citation analysis can be used to define disciplines and emerging specialties, as well as to determine the transdisciplinary or multidisciplinary nature of research programs and initiatives. The cited documents are linked together by the process of co-citation, which is analogous to the similarity metrics of word co-occurrence. To display the diversity of documents and communities in the systems thinking literature, document co-citation networks were created. Co-citation analysis of sources of publications was done, and it was found that out of 2,115 sources, 216 meet the threshold. A total of four clusters were found in the analysis.

**Figure 7:**
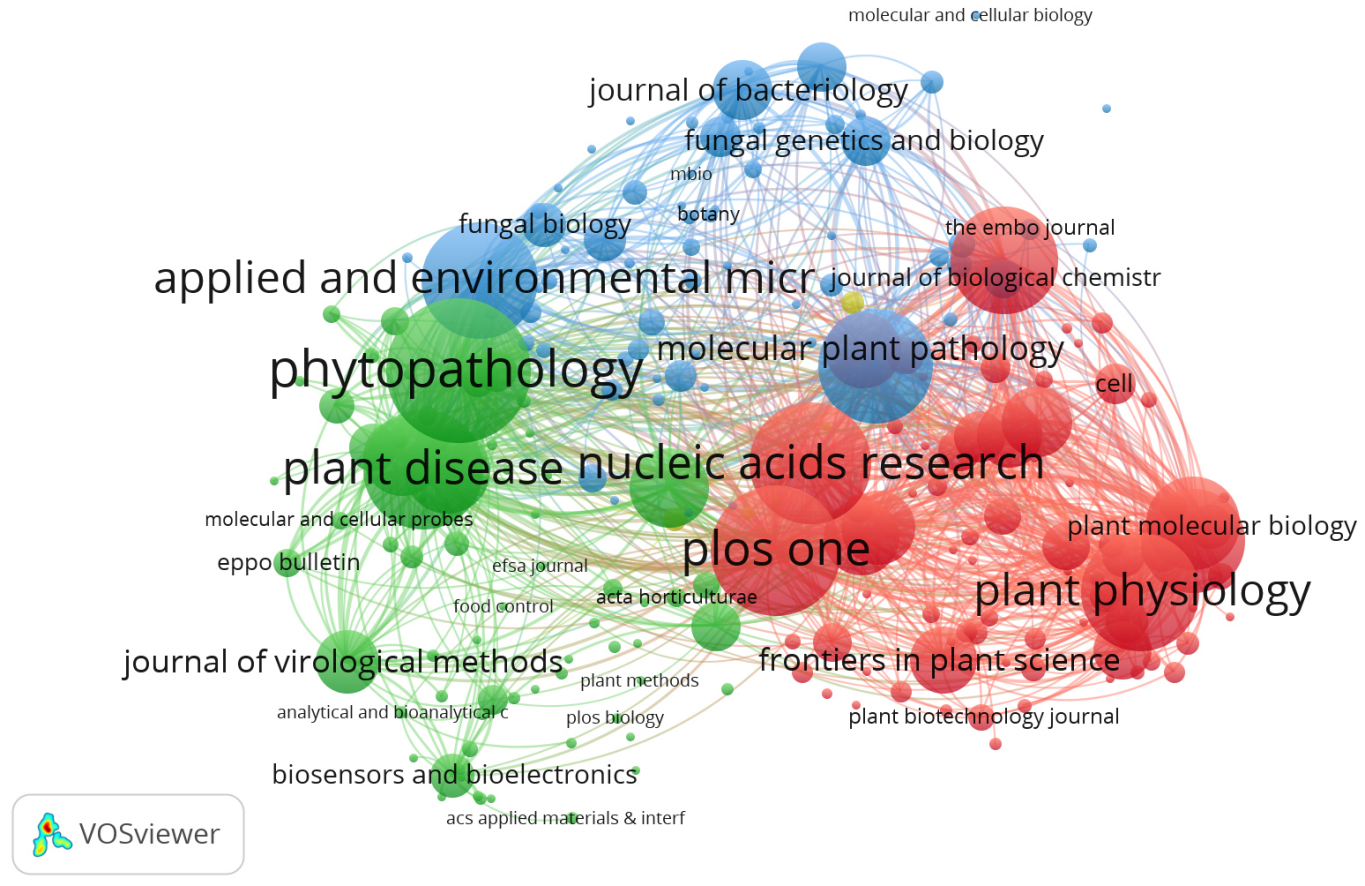
Co-citation analysis of sources. Source: VOSviewer.

From **Figure 7**, we can conclude that among the clusters, the highest number of citations and publications was found for the journals *Phytopathology*, *Plant Physiology*, *Plos One*, etc. These are the journals that cover the topic of real-time PCR technique of detection of plant pathogens.

By this co-citation network, we see how multiple sources recognize a common set and theme of documents (Braam et al., 1991). Therefore, future work would benefit from analysis of both cited and citing works to understand what ideas, findings, or experiments are being communicated and the meaning attributed to the co-cited documents. In addition, a survey of leading sources or analyzing keywords may reveal deeper patterns underlying co-citations (Small, 1977).

### 3.6 Keyword analysis

**Figure 8:**
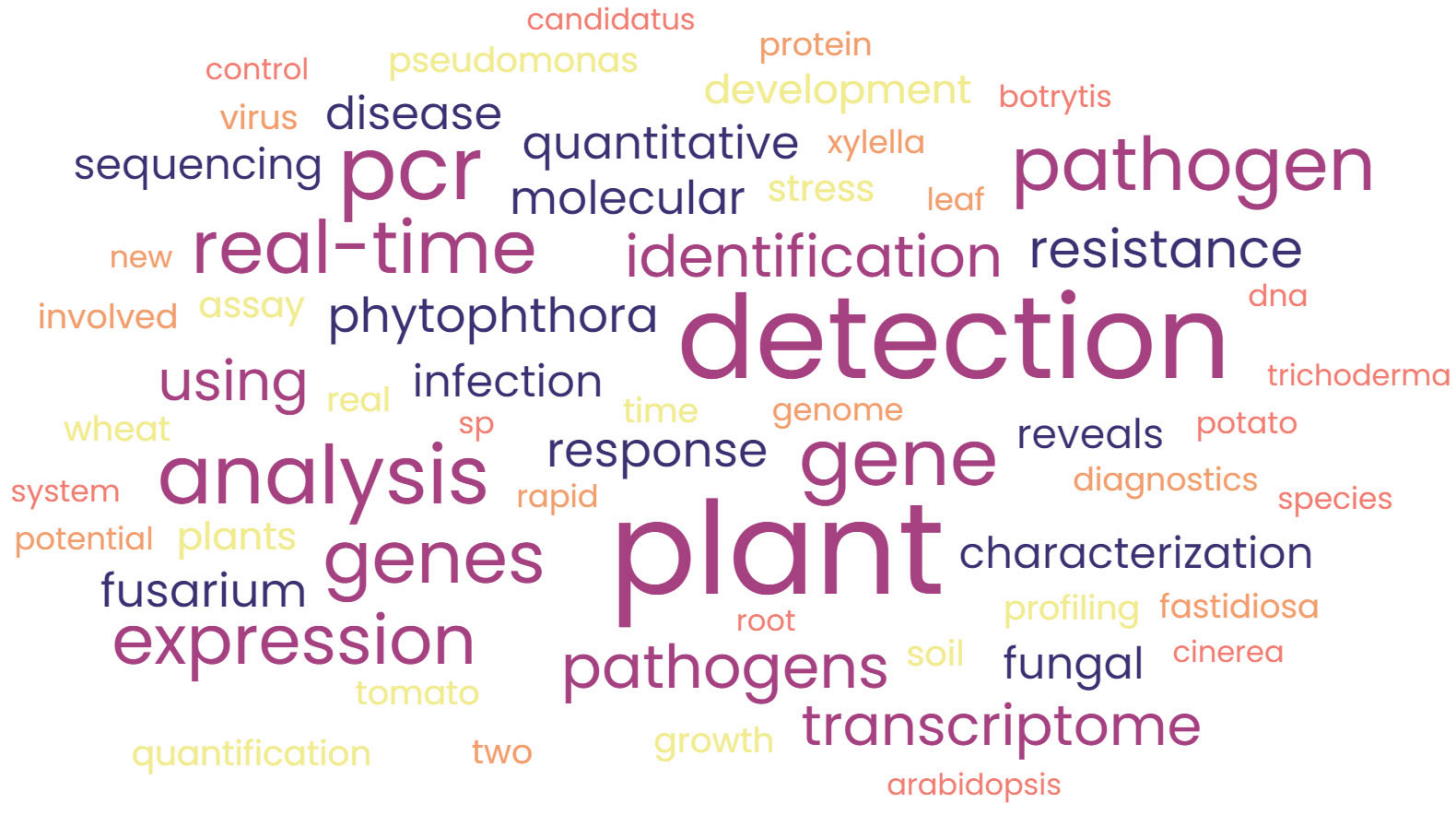
Word cloud of frequently used keywords.

Keyword analysis gives us an insight into the words that the authors feel is important in their papers. In this analysis, we conducted keyword analysis based on the words of the title chosen by the researchers in their manuscript. The reason behind this is that the Dimensions data retrieved do not particularly allow us to conduct keyword analysis. There are few advantages of using keyword analysis as it gives an idea about trending research topics. The analysis will also reveal the direction of research in the theme of study. The results of keyword analysis are presented as a word cloud in **Figure 8**.

The keyword analysis results clearly indicate that the studies focused on detection in plants only as “plant” was found to be the most highlighted word in the figure. It is to be noted that the other highlighted texts were “gene,” “*Fusarium*,” “*Phytophthora*,” “transcriptome,” etc., which depict the direction of trends in the research of nucleic acid–based pathogen detection.

## 4 Conclusion

This bibliometric analysis clearly depicted that articles on RT-PCR-based pathogen detection are consistently increasing. The exponential increase in publications related to the detection technique can be attributed to its versatility and usefulness in screening a large number of germplasms of horticultural and field crops. Additionally, the technique has been used extensively in functional validation studies of transcriptomic data, contributing to its widespread adoption and popularity in the field. A comprehensive analysis of articles indexed in the “Dimensions” database from 2001 to 2021 revealed that the United States and China were the top countries in terms of the number of publications related to this detection technique. This suggests that both countries have made significant contributions to the development and advancement of the technique and are leaders in the field. The findings of this analysis provide valuable insights into the global research landscape and highlight the importance of continued research and innovation in this area. The top journals were primarily *Plos One*, followed by *BMC Genomics* and *Phytopathology*. The most cited journal was the *European Journal of Plant Pathology*. From a future perspective, the multi-institutional and multidisciplinary relationship between different countries requires further strengthening. The changing pest and pathogen dynamics and rapid advancement of novel isothermally based detection tools will affect the crucial role of RT-PCR in plant disease diagnostics. This is an ongoing process, and with the advent of new software and the involvement of a more exhaustive database, the study can be further strengthened.

## Availability of data and materials

The datasets generated and analyzed in the current study are available in this manuscript.

## Data availability statement

The original contributions presented in the study are included in the article/supplementary material. Further inquiries can be directed to the corresponding authors.

## Author contributions

PL, ML, and RT: conceptualization, methodology, investigation, writing—original draft preparation. RK, MA, AAA, and AK: reviewing, analysis editing. All authors contributed to the article and approved the submitted version.
